# The quadruplex TaqMan MGB fluorescent quantitative PCR method for simultaneous detection of feline panleukopenia virus, feline herpesvirus 1, feline calicivirus and feline infectious peritonitis virus

**DOI:** 10.3389/fcimb.2025.1581946

**Published:** 2025-05-30

**Authors:** Haojie Wang, Lihong Xue, Longxi Wang, Yixuan Liu, Jianxing Chen, Yue Sun, Tongqing An, Hongyan Chen, Changqing Yu, Changyou Xia, He Zhang

**Affiliations:** ^1^ State Key Laboratory for Animal Disease Control and Prevention, Harbin Veterinary Research Institute, Chinese Academy of Agricultural Sciences, Harbin, China; ^2^ School of Advanced Agricultural Sciences, Yibin Vocational and Technical College, Yibin, China

**Keywords:** FPV, FHV-1, FCV, FIPV, quadruplex, TaqMan MGB fluorescent

## Abstract

**Background:**

Feline panleukopenia, feline calicivirus infection, feline viral rhinotracheitis, and feline infectious peritonitis are significant diseases that threaten feline health. The trend of mixed infections is increasing, and current diagnostic methods are limited in scope and unable to provide rapid, simultaneous detection of these diseases.

**Methods:**

Four groups of primers and probes targeting the *VP2* gene of Feline Panleukopenia virus (FPV), the *TK* gene of Feline Herpesvirus (FHV-1), the *ORF2* gene of Feline Calicivirus (FCV), and the *N* gene of Feline Infectious Peritonitis Virus (FIPV) were designed. After optimizing the concentrations of primers and probes and annealing temperature, a quadruplex TaqMan MGB fluorescent quantitative PCR method was established to concurrently detect these four pathogens. Recombinant plasmid standards were constructed to establish standard curves, and the sensitivity, specificity, reproducibility, and clinical application of the assay were evaluated.

**Results:**

The optimal final concentrations of primers for FPV, FHV-1, FCV, and FIPV were 0.08, 0.04, 0.06, and 0.12 μM, respectively, and the optimal final concentrations of probes were 0.08, 0.08, 0.12, and 0.12 μM, respectively. The best annealing temperature was 59°C. No cross-reaction was observed with common pathogens in infected cats. The minimal detection limits for recombinant plasmids of T-VP2, T-TK, T-ORF2, and T-N were 50.79, 53.21, 47.91 and 41.25 copies/μL, respectively. The R² values of standard curves are 0.994, 1.0, 0.998 and 0.999, respectively, and high amplification efficiencies of 105.05%, 96.28%, 98.82%, and 96.45%, respectively. The coefficient of variation for inter-batch and intra-batch tests ranged from 0.14 to 1.37%. Among 381 fecal samples from cats, the detection rates for FPV, FHV-1, FCV, and FIPV were 13.65% (52/381), 18.37% (70/381), 26.77% (102/381), and 9.71% (37/381), respectively, with a 100% agreement with previously reported methods and commercial kits.

**Conclusion:**

The sensitive, specific, high-throughput, quadruplex TaqMan MGB quantitative fluorescent quantitative PCR method was successfully established for the simultaneous detection of FPV, FHV-1, FCV, and FIPV.

## 1 Introduction

Cats are known for their gentle and independent nature, their ability to provide therapeutic companionship, and their strong emotional bond with humans, making them one of the most important companion animals. According to the *China Pet Industry White Paper*, the number of pet cats in 2024 is projected to reach 71.93 million, marking a 2.5% increase from 2023 and continuing an upward trend (Source Network). However, a large body of clinical data indicates that feline panleukopenia, feline viral rhinotracheitis, feline infectious peritonitis, and feline calicivirus infections have relatively high incidence rates among felines and are often associated with mixed infections, posing a serious threat to the health of global feline populations (Tasker et al., 2023; Longobardi et al., 2024; Liu et al., 2025; Nishisaka et al., 2025).

Feline panleukopenia, also known as feline distemper, feline parvovirus, or feline infectious enteritis, is a disease caused by Feline Panleukopenia virus (FPV), a member of the *Parvoviridae* family (Yang et al., 2024a). It primarily affects kittens under one year of age (Ye et al., 2022). FPV is a single-stranded DNA virus with a genome of approximately 5,200 base pairs, which includes two open reading frames that encode the non-structural proteins (NS1 and NS2) and structural proteins (VP1 and VP2) (Li et al., 2022). FPV can infect various animals, including felids (such as tigers, leopards, and lions) and mustelids (such as raccoons and ferrets) (Huang et al., 2022; Wang et al., 2022). The virus is primarily transmitted through feces, saliva, and other secretions, with infected cats serving as the main source of transmission (Yu et al., 2024). The disease is most common during the spring and summer months and can affect cats of all ages, with kittens particularly vulnerable (Pacini et al., 2023). The infection rate in kittens can be as high as 70%, and the fatality rate ranges from 45% to 60% (Xue et al., 2023a; Xue et al., 2023b). A study conducted in Yanji, China, in 2021–2022 reported a 33.75% FPV positivity rate (27/80) (Xue et al., 2023a).

Feline viral rhinotracheitis is a viral infectious disease caused by Feline Herpesvirus 1 (FHV-1), which primarily affects the respiratory and ocular systems of felids (Synowiec et al., 2023). FHV-1 is a member of the *Herpesviridae* family, subfamily *Alphaherpesvirinae*, and genus *Varicellovirus (* Yang et al., 2024b). The FPV genome is a double stranded DNA composed of a unique region (UL) approximately 99 kb in length and short fragments of approximately 27 kb (Cavalheiro et al., 2023). Maeda et al. classified FHV-1 into three genotypes: F2, C7301, and C7805 (Maeda et al., 1995). FHV-1 mainly infects felids, particularly kittens, and is widely distributed globally (Jiao et al., 2024). The virus is primarily transmitted through direct contact and aerosols and often co-infects with FCV, leading to symptoms such as respiratory distress and oral ulcers (Gaskell and Povey, 1982). Its incidence can reach 100%, with a mortality rate between 20% and 50% (Liu et al., 2022). A study conducted in Kunshan, China, in 2022–2023 reported a 21.5% FHV-1 positivity rate (43/200) in conjunctival and nasal swabs from cats exhibiting respiratory distress, conjunctivitis, and corneal ulcers (Kim et al., 2024).

Feline calicivirus (FCV) infection primarily manifests as oral ulcers, pneumonia, chronic gastritis, arthritis, and lameness (Wei et al., 2024). FCV, a member of the *Caliciviridae* family, is one of the most common viral pathogens in domestic cats worldwide (Duclos et al., 2024). FCV genome is a single stranded RNA about 7700 bp in length, contains three open reading frames (ORFs): ORF1, ORF2, and ORF3 (Wei et al., 2024). ORF2 encodes the capsid protein VP1, which contains six variable regions (A-F) (Zhou et al., 2021). Based on genetic evolution, FCV strains are categorized into Type I and Type II. FCV is distributed globally and can infect not only domestic cats but also other felid species (Mao et al., 2022). The virus primarily spreads through respiratory secretions and saliva and mainly affects kittens aged 7–84 days, with the highest susceptibility seen in those aged 56–84 days (Wei et al., 2024). FCV has a high incidence rate, but its fatality rate is usually low (Wei et al., 2024). A study conducted in Guangdong Province, China, from 2018 to 2022 reported a 28.9% FCV positivity rate in throat and nasal swabs from doubtful FCV-infected cats (152 samples) (Mao et al., 2022).

Feline infectious peritonitis is caused by Feline Coronavirus (FCoV) (Thayer et al., 2022). FCoV is a single-stranded RNA virus with a genome about 30000 bp in length, containing 11 ORFs (Gao et al., 2023). The virus particles have an envelope with a diameter ranging from 80 to 120 nm (Kennedy, 2020). Based on differences in the spike protein and serotype, FCoV can be classified into two types: Feline infectious peritonitis virus (FIPV) and Feline Enteric Coronavirus (FECV) (Bubenikova et al., 2020; Chen et al., 2023). FECV infection often causes mild or asymptomatic diarrhea, but 5%–12% of infected cats will become Feline infectious peritonitis due to mutations of FECV into FIPV, with a fatality rate approaching 100% in cats with Feline infectious peritonitis (Lewis et al., 2015). Studies suggest that 0.3% to 1.4% of feline deaths in veterinary clinics are attributed to Feline infectious peritonitis (Thayer et al., 2022). Currently, there is no effective vaccine available to prevent this disease.

Common laboratory detection methods for FPV, FHV-1, FIPV, and FCV include viral isolation, serological tests, and molecular biological techniques (Liu et al., 2022; He et al., 2024). Virus isolation is complex, time-consuming, and difficult to implement clinically (Wang et al., 2024c). Serological tests may lead to false-negative owing to the stage of viral infection and the host’s immune status (Wang et al., 2024b). PCR technology, especially fluorescence quantitative PCR (qPCR), offers high specificity, sensitivity, and stability, and it can also quantify target genes (Wang et al., 2024a). However, most qPCR methods for detecting these four viruses are singleplex, limiting the ability to detect multiple pathogens simultaneously (Zhang et al., 2019; Liu et al., 2024). Therefore, the construction of a quadruplex TaqMan MGB qPCR method capable of simultaneously detecting FPV, FHV, FIPV, and FCV can not only improve diagnostic efficiency but also reduce testing costs.

In summary, the above four viruses have high prevalence rates among felines, causing significant harm, and are easy to mixed or secondary infections, become clinical diagnosis difficult. Additionally, vaccines have been developed for some of these viruses, such as the trivalent inactivated vaccine for feline viral rhinotracheitis, calicivirus disease, and panleukopenia (RPVF0304, RPVF0207, and RPVF0110 strains), as well as vaccines for feline rhinotracheitis, calicivirus, and panleukopenia (CP2, CC3, and VP2 proteins) (Source Network). However, there is an urgent need for rapid, efficient, sensitive, and specific diagnostic methods to screen antigen-negative cats, as these four viruses must be detected in the breeding of specific-pathogen-free (SPF) cats. In this study, four sets of specific primers and probes the *VP2* gene of FPV, the *TK* gene of FHV-1, the *N* gene of FIPV, and the *ORF2* gene of FCV were designed to establish a quadruplex TaqMan MGB fluorescent quantitative PCR method. The goal of this method is to provide rapid, sensitive, and specific detection of these four viruses, offering technical support for early diagnosis, timely treatment, vaccine evaluation, and SPF cats breeding, ultimately contributing to the improvement of feline health and reducing the risk of disease transmission.

## 2 Materials and methods

### 2.1 Nucleic acids of virus and bacterium

The nucleic acids of Feline Parvovirus (FPV), Feline Herpesvirus 1 (FHV-1), Feline Infectious Peritonitis Virus (FIPV), Feline Calicivirus (FCV), *Salmonella*, *Pasteurella multocida*, *Staphylococcus aureus*, Feline Leukemia Virus (FeLV), and Feline Immunodeficiency Virus (FIV) were stored in our laboratory. The clinical samples (nasal, oral swabs, and feces) of 381 cats were collected from some cat farms in Hebei Province, which were from healthy cats, cats with respiratory symptoms, or cats with diarrhea. It is imperative to underscore that no additional harm or intervention was imposed on the animals involved in this study. Given the nature of our research, the Institutional Review Board of the Harbin Veterinary Research Institute has determined that this study is exempt from the requirement for ethical review or approval.

### 2.2 Synthesis of plasmid standards and design of primers and probes

Based on the gene sequences registered in NCBI, four groups of specific primers and probes were designed targeting the *VP2* gene of FPV (Accession No: X55115.1), the *TK* gene of FHV-1 (Accession No: MT813102.1), the *ORF2* gene of FCV (Accession No: PP928983.1), and the *N* gene of FIPV (Accession No: KY566183.1) using SnapGene software and PrimerSelect software. The primers and probes were synthesized by Genscript (**Table 1**). The conserved sequences of the target genes were downloaded from NCBI, and then sent to Genscript for plasmid construction. The plasmids were cloned into the pMD-18T vector and named T-VP2, T-TK, T-ORF2, and T-N. The plasmid concentrations were determined, and the copy numbers were calculated using the standard formula.

**Table 1:** Sequences of primers and probes for the quadruplex TaqMan MGB fluorescent quantitative PCR method.

Pathogens	Gene	Sequence(5′-3′)	Product size
FPV	*VP2*	F: ATGAGACCAGCTGAGGTTGGTT R: CCCCGTCCTGCTGCAATA Probe: FAM-CGTCTACACAAGGGC-MGB	105 bp
FHV-1	*TK*	F: GATTTGCCGCACCATACCTT R: CAGAGAGGCGAGAGGGTGTCT Probe: NED-TTTTACATTCCAGACTATCCAC-MGB	125 bp
FCV	*ORF2*	F: GCATTGGGATGAAGCAGGTAA R: CCATCATCCGCTTCCAATCT Probe: TAMRA-ATCTTCCAACCACACCC-MGB	126 bp
FIPV	*N*	F: TTGTCAAGGGTCAGCGTAAGG R: GCAACCCAGAAGACACCATCA Probe: ROX-CTTCCTGAGAGGTGGTTC-MGB	115

The recombinant plasmid standards copies/µL = (6.02×10^23^) × (X^*^ ng/µL × 10^−9^)/constructed plasmid length (bp) × 660.

* X: Standard plasmid concentration

### 2.3 Optimization of annealing temperature, primer, and probe concentrations

The four recombinant plasmid standards, T-VP2, T-TK, T-ORF2, and T-N, were mixed in equal proportions and used as templates. Four groups of primers and probes were employed for fluorescent quantitative TaqMan MGB PCR amplification in the same system. The annealing temperature (58, 59, 60, 61, 62, 63°C), four primer concentrations (final concentrations of 0.01-0.5 μM), and four probe concentrations (final concentrations of 0.01-0.5 μM) were optimized using the matrix assay. In addition, compare the effects of different cycle numbers (35, 40, 45 and 50) on amplification. The annealing temperature, sprimer, and probe concentrations were selected according to the smallest Ct values and maximum fluorescence signals to establish the optimal conditions for quadruplex TaqMan MGB fluorescent quantitative PCR method. The above experiments were repeated three times.

### 2.4 Construction of standard curves for quadruplex TaqMan MGB qPCR method

The four recombinant plasmid standards, T-VP2, T-TK, T-ORF2, and T-N, were each adjusted to 4 × 10^10^ copies/μL. After 10-fold serial dilution, they were mixed in equal proportions. Each concentration was repeated three times. Using the established quadruplex fluorescence quantitative TaqMan MGB PCR method for amplification, and standard curve was constructed with Ct values on the y-axis and the logarithm of template copy numbers on the x-axis.

### 2.5 Sensitivity test

The four recombinant plasmid standards, T-VP2, T-TK, T-ORF2 and T-N, were mixed in equal proportions after 10-fold serial dilution. The plasmid mixture with final concentrations ranging from 1 × 10^7^ to 1 × 10^0^ copies/μL was used as the template (Repeat each gradient three times). The Quadruplex TaqMan MGB qPCR assay amplification was performed using the established method. Sensitivity of the method was evaluated by probit regression analysis (Ma et al., 2024).

### 2.6 Specificity test

DNA/RNA (approximately 50 ng/μL) from FPV, FHV-1, FIPV, FCV, *Salmonella*, *Pasteurella multocida*, *Staphylococcus aureus*, FeLV, and FIV were used as templates. The four recombinant plasmid standard mixtures were used as positive controls, and sterilized double-distilled water was used as the negative control. The established quadruplex TaqMan MGB qPCR assay was employed to assess the specificity of the method.

### 2.7 Repeatability test

The four recombinant plasmid standards, T-VP2, T-TK, T-ORF2, and T-N, were mixed in equal proportions after 10-fold serial dilution. The plasmid mixtures at final concentrations of 1 × 10^7^ copies/μL, 1 × 10^5^ copies/μL, and 1 × 10^3^ copies/μL were used as templates. Triple amplification was performed for each sample in an intra-assay repeatability test. The plasmid mixtures from different time points at the three concentrations were used as templates to conduct an inter-assay repeatability test, with each sample repeated three times. The coefficient of variation (CV) for intra-assay and inter-assay repeatability was calculated to assess the repeatability of the method. In addition, we used different experimenters and fluorescence quantitative PCR machines to test the stability of the method.

### 2.8 Detection of clinical samples

From August to December 2024, 381 clinical samples (swabs, feces, etc.) were collected from a cat farm in Hebei Province for testing. Resuspend the sample in sterile PBS buffer, centrifuge at 5000rpm for 3 minutes, and collect the supernatant. Extract DNA/RNA using VAMNE Magnetic Pathogen DNA/RNA Kit (Vazyme, China). The extracted nucleic acid is stored at -20°C or -80°C for future use. Then, the samples were simultaneously tested using the quadruplex TaqMan TaqMan MGB qPCR, reported methods, and commercial kits to verify the accuracy of this method (Cao et al., 2021; Zou et al., 2022; Liu et al., 2024).

## 3 Results and analysis

### 3.1 Determination of the optimal conditions for quadruplex TaqMan MGB qPCR method

The optimization results from the matrix method showed that the optimal reaction system for quadruplex TaqMan MGB fluorescent quantitative PCR was 25 μL: 12.5 μL of 2×Fast One Step Probe RT-qPCR Mix (Takara, China), 0.2 μL of forward and reverse primers for FPV, 0.2 μL of probe, 0.1 μL of forward and reverse primers for FHV-1, 0.2 μL of probe, 0.15 μL of forward and reverse primers for FCV, 0.3 μL of probe, 0.3 μL of forward and reverse primers for FIPV, 0.25 μL of probe (The working concentrations of primers and probes were 10 μ M), 2 μL of nucleic acids, and 7.6 μL of sterilized double-distilled water. Using different cycle numbers for amplification, the results showed that when the cycle number was 35, the amplification was insufficient; When the number of cycles is 45 and 50, there is non-specific amplification. So we chose the optimal number of cycles as 40. The amplification program was as follows: 52°C for 5 minutes, 95°C for 10 seconds, 95°C for 5 seconds, 59°C for 10 seconds, for 40 cycles, with fluorescence signal collection during each cycle.

### 3.2 Construction of standard curves for quadruplex TaqMan MGB fluorescent quantitative PCR method

**Figure 1:**
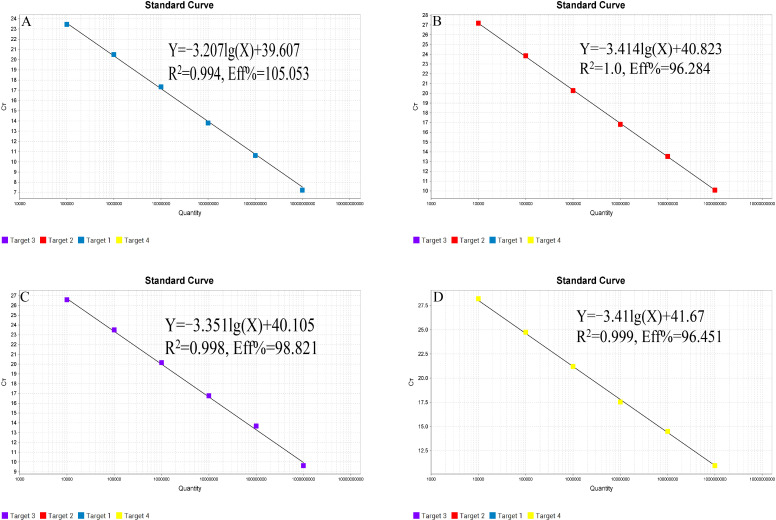
Standard curves of quadruplex TaqMan MGB qPCR method. **(A)** FPV; **(B)** FHV-1; **(C)** FCV; **(D)** FIPV.

The recombinant plasmid standards, T-VP2, T-TK, T-ORF2, and T-N, were mixed in equal proportions after 10-fold serial dilution. Amplification was performed using the method established in this study. A standard curve was constructed with Ct values on the y-axis and the logarithm of template copy numbers on the x-axis (**Figure 1**). For FPV, the standard curve was constructed with concentrations ranging from 1 × 10^10^ to 1 × 10^5^ copies/μL; for FHV-1, FCV, and FIPV, the standard curves were constructed with concentrations ranging from 1 × 10^9^ to 1 × 10^4^ copies/μL. The equations for the standard curves were as follows: FPV: Y = -3.207lg(X) + 39.607, R² = 0.994, EFF% = 105.053; FHV-1: Y = -3.414lg(X) + 40.823, R² = 1.0, EFF% = 96.284; FCV: Y = -3.351lg(X) + 40.105, R² = 0.998, EFF% = 98.821; FIPV: Y = -3.41lg(X) + 41.67, R² = 0.999, EFF% = 96.451%.

### 3.3 Sensitivity results

**Figure 2:**
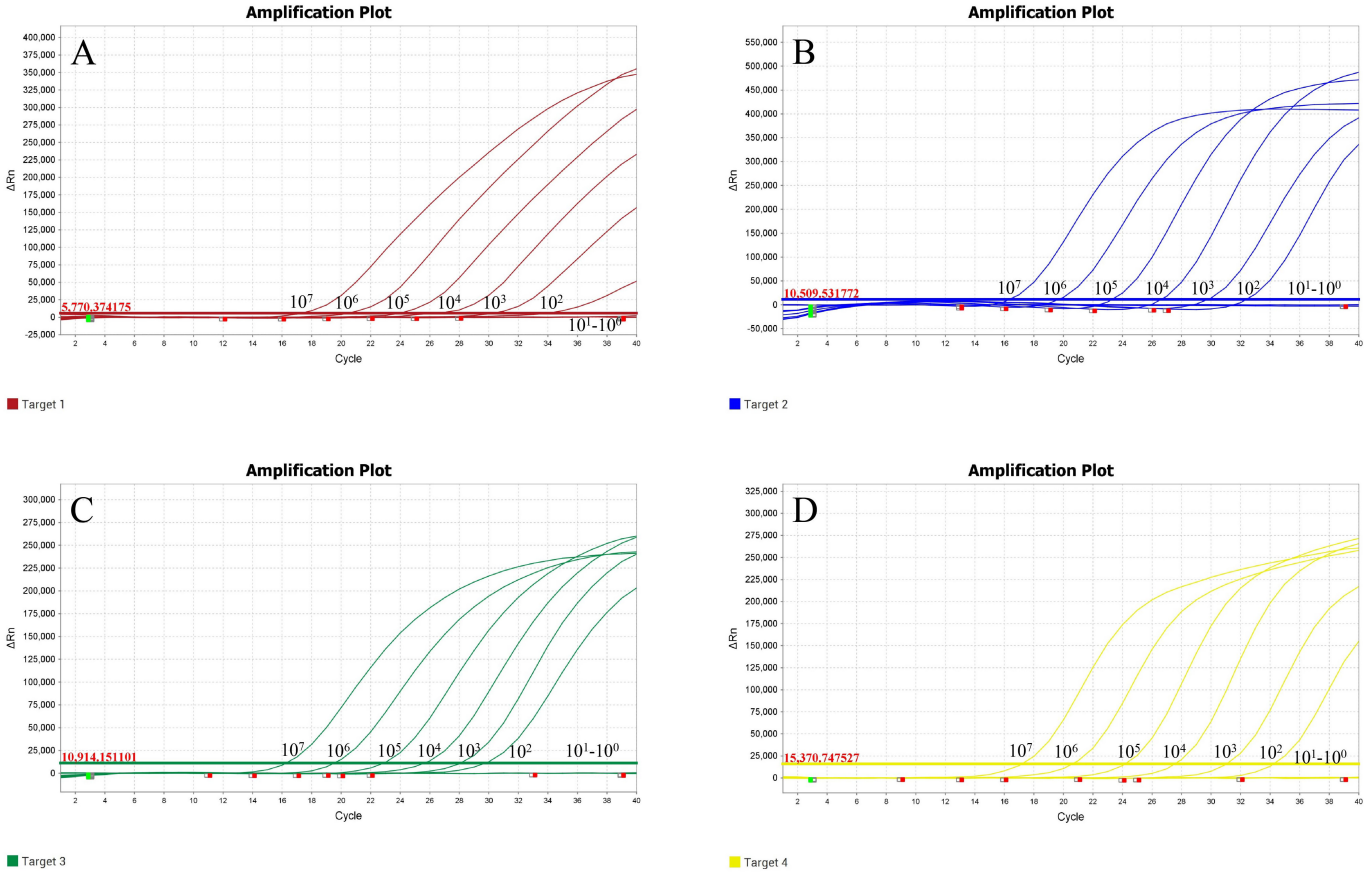
Sensitivity test results of the quadruplex TaqMan MGB qPCR method. **(A)** FPV; **(B)** FHV-1; **(C)** FCV; **(D)** FIPV.

**Figure 3:**
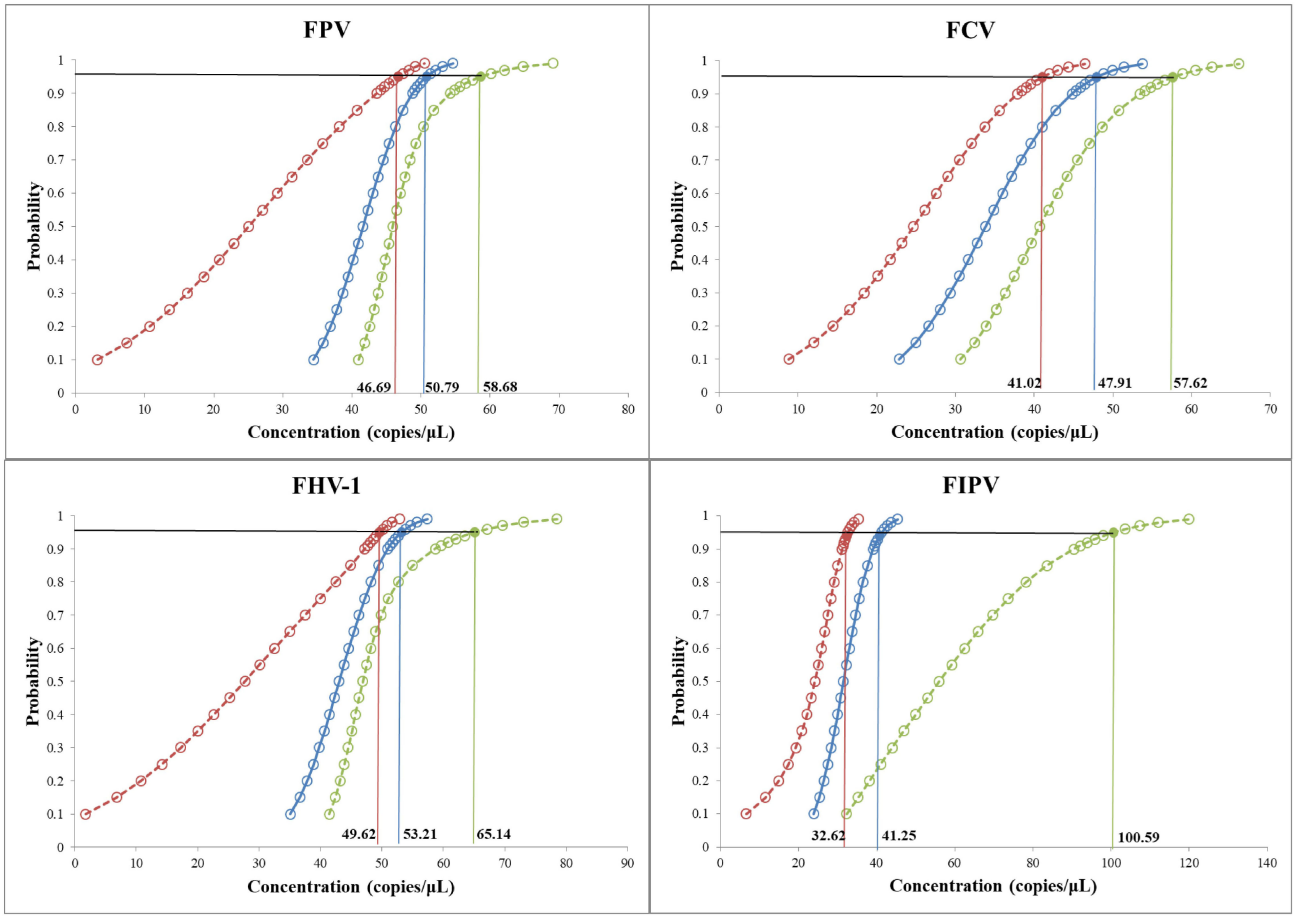
Probability regression analysis of the quadruplex TaqMan MGB qPCR method.

Plasmid standards T-VP2, T-TK, T-ORF2, and T-N were diluted in a 10-fold series and mixed in equal proportions (final concentrations ranging from 1 × 10^10^ copies/μL to 1 × 10^0^ copies/μL) to serve as templates for the sensitivity test of the quadruplex TaqMan MGB qPCR method. The results showed that the lower detection limit of the quadruplex TaqMan MGB qPCR system reached 100 copies/μL, demonstrating that the method retains a certain level of sensitivity even in the competitive inhibitory quadruplex reaction system (**Figure 2**). Additionally, we used probability regression analysis to determine the minimum detection limit of this method. The quadruplex TaqMan MGB qPCR method was used to detect T-VP2, T-TK, T-ORF2, and T-N plasmid mixtures at concentrations of 400 copies/μL, 200 copies/μL, 100 copies/μL, 50 copies/μL, 25 copies/μL, and 12.5 copies/μL. The results, including average Ct values and hit rates, are shown in **Table 2**. The minimum detection limits for FPV, FHV-1, FCV, and FIPV were 50.79 (95% confidence interval: 46.69–58.68), 53.21 (95% confidence interval: 49.62–65.14), 47.91 (95% confidence interval: 41.02–57.62), and 41.25 (95% confidence interval: 32.62–100.59), respectively (**Figure 3**). When the Ct values of FPV, FHV-1, and FCV are less than 34 and the amplification curves are smooth, the above pathogens are considered positive; When the Ct value of FIPV is less than 36 and the amplification curve is smooth, FIPV is considered positive. When the Ct value of the four pathogens is 40 or no value, it is judged as negative. When the amplified Ct value is greater than the critical value and less than 40, it is considered suspicious and double retesting is recommended.

**Table 2:** Average Ct values and hit rates of plasmid standards at various dilution levels.

Recombinant plasmids	Concentrations(copies/μL)	samples	the Quadruplex Fluorescent Quantitative PCR Method	
			Ct(average)	Detection rate(%)
T-VP2	400	30	31.57	100
	200	30	32.61	100
	100	30	33.28	100
	50	30	34.87	93.33
	25	30	Undetermined	0
	12.5	30	Undetermined	0
T-TK	400	30	32.07	100
	200	30	32.81	100
	100	30	34.02	100
	50	30	35.85	86.67
	25	30	Undetermined	0
	12.5	30	Undetermined	0
T-ORF2	400	30	31.15	100
	200	30	32.33	100
	100	30	33.51	100
	50	30	35.11	96.67
	25	30	Undetermined	16.67
	12.5	30	Undetermined	0
T-N	400	30	32.81	100
	200	30	33.46	100
	100	30	34.94	100
	50	30	35.79	100
	25	30	37.11	13.33
	12.5	30	Undetermined	0

### 3.4 Specificity results

**Figure 4:**
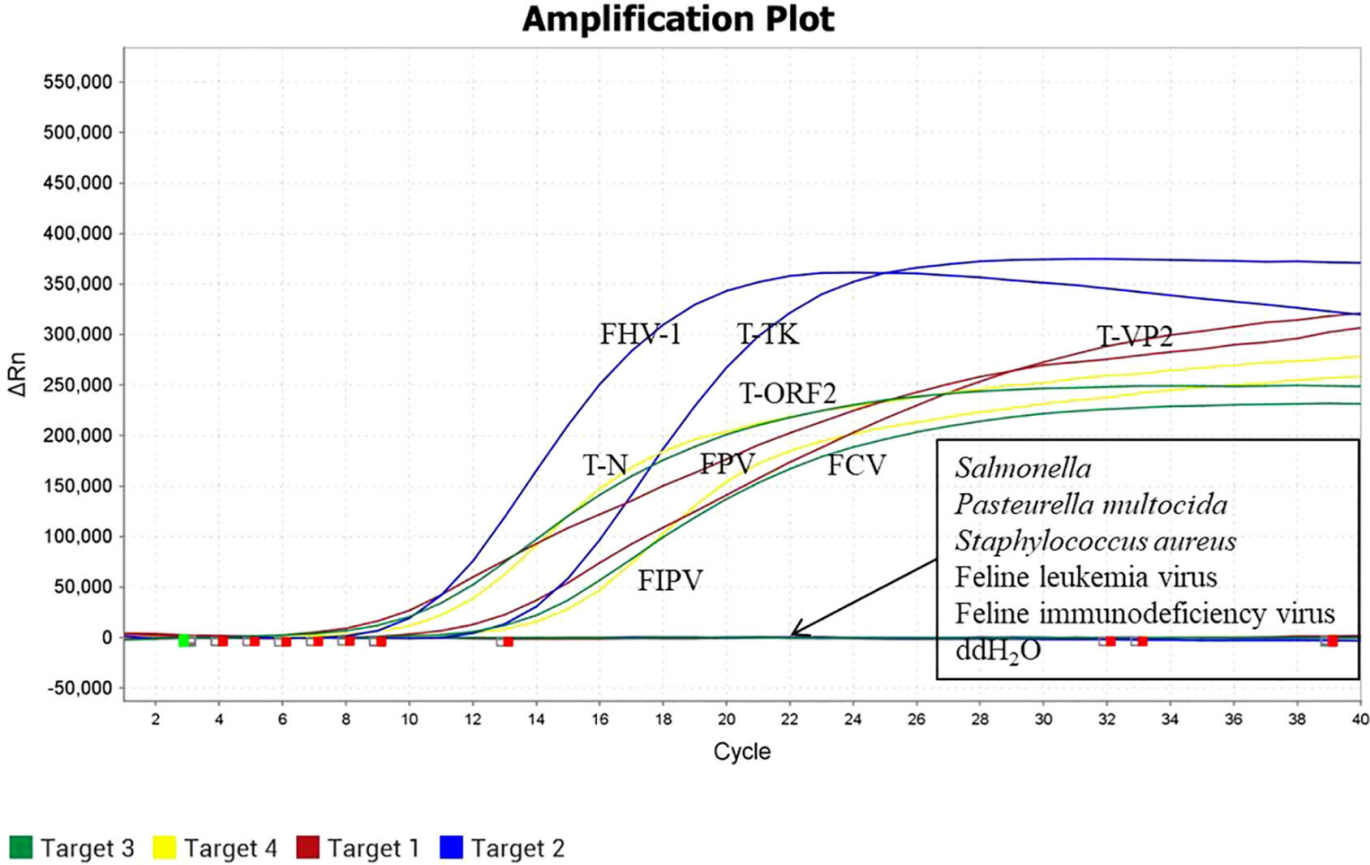
Specificity test results of the four fluorescent quantitative TaqMan MGB PCR methods.

The quadruplex TaqMan MGB qPCR method was used to detect the DNA/RNA (approximately 50 ng/μL) of FPV, FHV-1, FIPV, FCV, *Salmonella*, *Pasteurella multocida*, *Staphylococcus aureus*, FeLV, FIV. The results showed that the nucleic acids of FPV, FHV-1, FIPV and FCV were specifically amplified, while no amplification was observed for the nucleic acids of other viruses or bacteria that infect cats (**Figure 4**). These findings indicate that the quadruplex TaqMan MGB qPCR method has high specificity.

### 3.5 Reproducibility results

Reproducibility tests, both intra-group and inter-group, were performed using the quadruplex TaqMan MGB qPCR method on plasmid standard mixtures at four concentrations (1×10^7^, 1×10^5^, and 1×10³ copies/μL). The results showed that the coefficient of variation (CV) of Ct values for both intra-group and inter-group tests was less than 1.5% (**Table 3**), indicating that the method has good reproducibility. This method has good stability among different experimenters and fluorescence quantitative PCR machines.

**Table 3:** Reproducibility test results of the quadruplex real time quantitative TaqMan MGB qPCR method.

Standard plasmid	Concentration of template (copies/μL)	Intra-coefficient of variation		Inter-coefficient of variation	
		X ± SD	CV (%)	X ± SD	CV (%)
T-VP2	10^7^	17.138 ± 0.037	0.21	17.084 ± 0.024	0.14
	10^5^	23.642 ± 0.101	0.43	23.691 ± 0.050	0.21
	10^3^	29.994± 0.114	0.38	30.011 ± 0.094	0.31
T-TK	10^7^	16.911 ± 0.232	1.37	16.563 ± 0.072	0.43
	10^5^	23.618 ± 0.098	0.41	23.712 ± 0.127	0.54
	10^3^	30.285 ± 0.078	0.26	30.331 ± 0.113	0.37
T-ORF2	10^7^	16.756 ± 0.043	0.26	16.592 ± 0.064	0.39
	10^5^	23.410 ± 0.103	0.44	23.613 ± 0.191	0.81
	10^3^	30.044 ± 0.214	0.71	29.989 ± 0.134	0.45
T-N	10^7^	17.808 ± 0.101	0.57	18.011 ± 0.043	0.24
	10^5^	24.518 ± 0.283	1.15	24.566 ± 0.144	0.59
	10^3^	31.639 ± 0.157	0.50	31.327 ± 0.367	1.17

### 3.6 Detection results of clinical samples

**Figure 5:**
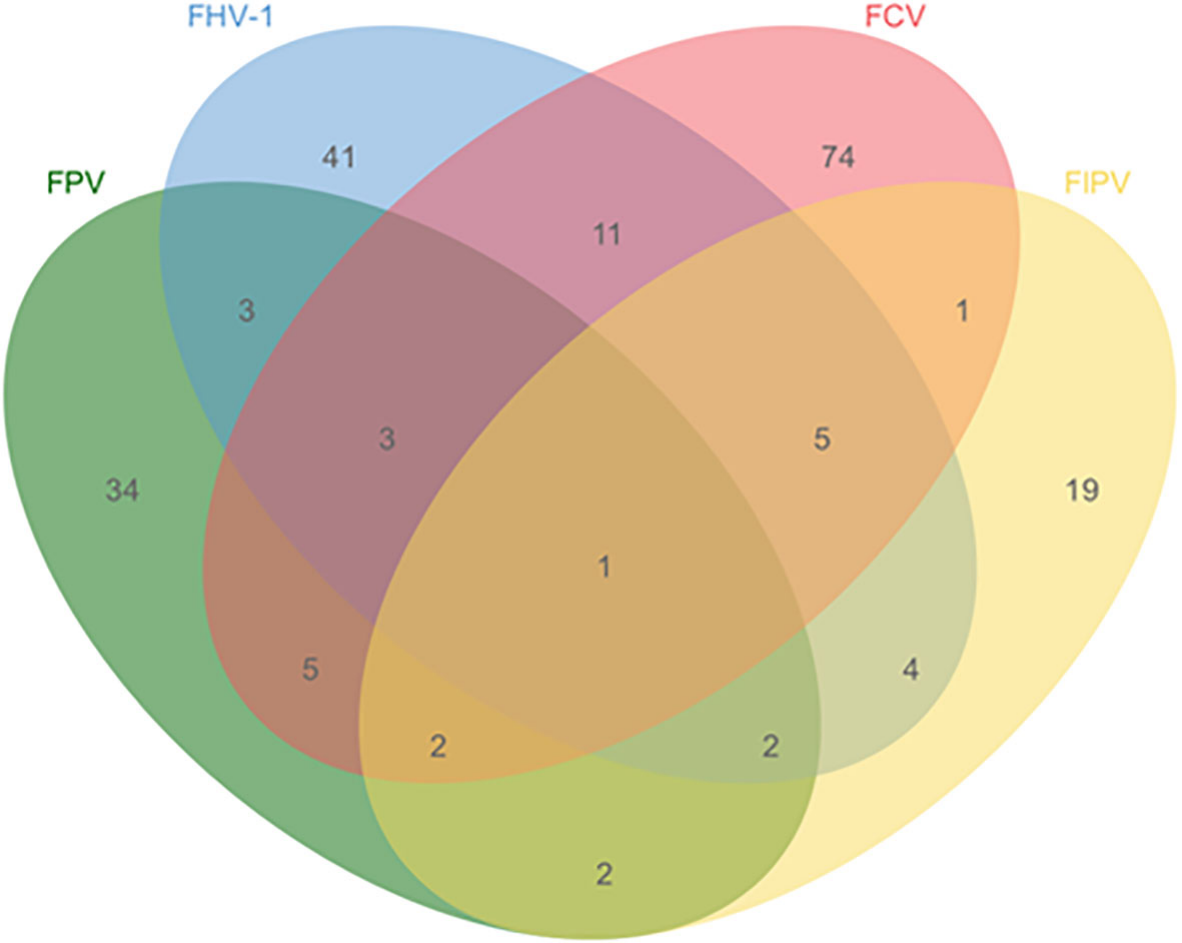
Single and mixed iInfections in 208 positive samples.

The quadruplex TaqMan MGB qPCR method was used to test 381 clinical cat clinical specimens. The results showed that the detection rates were 13.65% (52/381) for FPV, 18.37% (70/381) for FHV-1, 26.77% (102/381) for FCV, and 9.71% (37/381) for FIPV, with an overall positive rate of 54.59% (208/381) and a negative rate of 45.41% (173/381). The mixed infection situation is shown in **Figure 5**. Additionally, these samples were tested using previously reported methods and commercial kits. The results indicated that the detection outcomes from both assays were consistent with those obtained using the method established, with a 100% concordance rate between the three methods. This demonstrates that the assay can be applied to the detection of clinical samples.

## 4 Discussion

Cats are one of the most important companion animals to humans, yet diseases significantly impact their health. In some prosperous countries, the incidence of feline infectious diseases is prevented through vaccination and sanitation measures, whereas in developing countries, due to limited prevention and control technologies, the incidence of these diseases remains high (Cao et al., 2021). Among many diseases, FPV, FHV, FCV, and FIPV are the most common infectious diseases in felines (Xue et al., 2023b; Liu et al., 2025). These pathogens are highly contagious and cause severe gastrointestinal and respiratory symptoms, with a high mortality rate in kittens. Clinically, many newly adopted cats or those with suspected symptoms require screening for multiple infectious diseases to avoid misdiagnosis. The most commonly used detection method is the colloidal gold strip test, but this method has low sensitivity and requires subjective judgment by veterinary staff (Zhang et al., 2019; Ye et al., 2022). For cats with low viral loads, faint test lines on the strip may lead to inconclusive results, resulting in potential misdiagnosis and doubts from clients. Therefore, establishing a sensitive, specific, and efficient method to simultaneously differentiate FPV, FHV, FCV, and FIPV is crucial for developing targeted prevention and treatment strategies.

We targeted the *VP2* gene of FPV, the *TK* gene of FHV-1, the *ORF2* gene of FCV, and the *N* gene of FIPV, and established a specific, sensitive, accurate, and high-throughput quadruplex TaqMan MGB qPCR method that can tandem distinguish these four viruses. Optimization of qPCR conditions is a critical step in establishing this method. The quadruplex TaqMan MGB fluorescent quantitative PCR is favored because it can detect multiple target sequences in a single experiment. However, as the number of primers and probes increases, there is greater potential for interference, which could lead to unsmooth curves or inhibition of amplification. The selection of fluorescent groups is also critical; these need to have non-overlapping wavelengths that can be distinctly separated. Therefore, probes with different wavelength reporter groups were designed at the 5’ end, ensuring that each probe’s fluorescence signal could be independently identified using specific filters or detection channels, which avoids spectral overlap and reduces non-specific amplification or background noise, thereby enhancing specificity and the reliability of results. We optimized the reaction conditions using both matrix method. The optimal final concentrations of primers for FPV, FHV-1, FCV, and FIPV were 0.08, 0.04, 0.06, and 0.12 μM, respectively. The optimal final concentrations of probes were 0.08, 0.08, 0.12, and 0.12 μM, and through system optimization, we minimized interference between primers and probes.

This study used TaqMan MGB qPCR, which employs shorter probes than conventional TaqMan probes, with a binding moiety at the 3’ end that inserts into the minor groove of DNA. This increases the annealing temperature of the probes and fixities the probe-target complex, thereby improving sensitivity and specificity. The minimum detection limits for the quadruplex TaqMan MGB qPCR method were 50.79, 53.21, 47.91, and 41.25 copies for FPV, FHV-1, FCV, and FIPV, respectively. The sensitivity for detecting FPV, FHV-1, and FCV is slightly higher or comparable to that of the FPV (*VP2*), FHV-1 (*TK*), and FCV (*ORF2*) multiplex fluorescent quantitative PCR method established by Cao N et al. (50 copies/reaction) (Cao et al., 2021). The FPV (*VP2*) nanoPCR method established by Xue H et al. has a sensitivity of 7.97 × 10^2^ copies/μL, while the sensitivity of our method is 100 times greater (Xue et al., 2023b). The dual SYBR Green I quantitative PCR method for FCoV (*5’UTR*) and FPV (*VP2*) established by Sun L et al. has detection limits of 47.4 and 77.7 copies/μL for FPV and FCoV, respectively, which are lower than our method’s sensitivity (Sun et al., 2021). Moreover, the SYBR Green I quantitative PCR method is prone to non-specific amplification, leading to false positives. The triplex fluorescent quantitative PCR method established by Liu Y et al. for detecting SARS-CoV-2 (*N*), CCoV (*S*), and FIP (*N*) had sensitivities of 21.83, 17.25, and 9.25 copies/μL, respectively (Liu et al., 2024). While our method’s sensitivity is slightly lower, it targets more pathogens, and the sensitivity of fluorescent quantitative PCR tends to decrease as the number of targets increases. Additionally, the quadruplex TaqMan MGB qPCR method established here can specifically detect FPV, FHV-1, FCV, and FIPV without cross-reactivity with other bacterial or viral infections in cats, indicating strong specificity.

Stability in fluorescent quantitative PCR is a key factor in ensuring accurate results. The reproducibility of quantitative PCR experiments is influenced by DNA polymerase activity, template DNA quality, and operational techniques. To ensure high reproducibility, it is important to use high-quality, consistently active DNA polymerase and to store template DNA at -20 or -80°C to avoid repeated freeze-thaw. During operation, sterile protocols should be followed, and DNA-free reagents should be used. It is also recommended to premix enzymes, primers, probes, and deionized water before adding template DNA and mixing thoroughly to minimize contamination and improve consistency. Using high-precision pipettes or increasing the sample volume can further improve accuracy and ensure stable and reliable results. The quadruplex TaqMan MGB qPCR method demonstrated good reproducibility, with both inter- and intra-batch variation coefficients below 2%. Clinical testing of 381 samples showed positive detection rates of 13.65% (52/381) for FPV, 18.37% (70/381) for FHV-1, 26.77% (102/381) for FCV, and 9.71% (37/381) for FIPV. These results were consistent with current reported prevalences of these pathogens, and mixed infections are becoming more common, indicating the need for prevention and control of feline diseases.

## 5 Conclusion

In this study, Specially sensitive primers and probes were designed based on the conserved and specific sequences of FPV, FHV-1, FCV, and FIPV. After systematic optimization of reaction conditions and system parameters, a quadruplex TaqMan MGB qPCR assay was established. This assay revealed good linearity of standard curves, high amplification efficiency, high sensitivity, strong specificity, good reproducibility, and high accuracy. It can supply technical support for the diagnosis and prevention of FPV, FHV-1, FCV and FIPV.

## Data Availability

The original contributions presented in the study are included in the article/supplementary material. Further inquiries can be directed to the corresponding authors.
